# Associations of musculoskeletal disorders with occupational stress and mental health among coal miners in Xinjiang, China: a cross-sectional study

**DOI:** 10.1186/s12889-021-11379-3

**Published:** 2021-07-06

**Authors:** Xue Li, Xu Yang, Xuemei Sun, Qiaoyun Xue, Xiaofan Ma, Jiwen Liu

**Affiliations:** 1 Department of Public Health, Xinjiang Medical University, Ürümqi, 830011 China; 2 Xiangya School of Public Health, Central South University, Changsha, 410008 China; 3 Department of Infection, First Affiliated Hospital of Xinjiang Medical University, Ürümqi, 830054 China

**Keywords:** Mental health, Occupational stress, Musculoskeletal disorders, Bayesian network

## Abstract

**Background:**

Musculoskeletal disorders (MSDs), a common type of occupational diseases, have become the main cause of absenteeism and early retirement in the occupational population, as well as a major risk factor for occupational disability. The purpose of this study was to investigate the effects of occupational stress and mental health on MSDs in coal miners in Xinjiang, China, to provide a theoretical basis for reducing the incidence of MSDs in coal miners and improving their physical and mental health.

**Methods:**

In this study, stratified cluster random sampling was used to randomly select six coal mining companies in Xinjiang, and 1675 coal miners were surveyed by questionnaire. The status of occupational stress, mental health and MSDs among coal miners was investigated by means of the Effort–Reward Imbalance questionnaire (ERI), Symptom Checklist-90(SCL-90), and Musculoskeletal disorders scale (MSDs) questionnaire.

**Results:**

The prevalence of MSDs among coal miners was higher, and there were statistical differences among different sexes, ages, working years, shifts, types of work, educational level and monthly income (*P* < 0.001). The prevalence of MSDs in different body parts in the occupational stress group and mental disorder group was higher than that in the normal group. The results of multivariate logistic regression analysis showed that females had an increased risk of developing MSDs (*OR* = 2.23, 95% *CI.*:1.50,3.33). The risk of MSDs was higher with age < 30 years (30-39 years,*OR* = 2.39, 95% *CI*.,1.68,3.40; 40-49 years,*OR* = 2.15, 95% *CI.*:1.52,3.04; 50-60 years:*OR* = 3.25, 95% *CI*.:2.09,5.07), and the longer the working years, the higher the risk of MSDs (*OR* = 1.90, 95% *CI*.:1.38,2.62). The two shifts group (*OR* = 2.18, 95% *CI.*:1.59,2.98) had an increased risk of developing MSDs compared with the fixed day shift group. The risk of MSDs was lower in heading drivers (*OR* = 0.41, 95% *CI.*:0.29,0.60,) and transport workers (*OR* = 0.30, 95% *CI.*:0.20,0.43). The higher the education level, the lower the risk of MSDs (high school: *OR* = 0.46, 95% *CI.*:0.34,0.62, junior college and above: *OR* = 0.12, 95% *CI.*:0.08,0.17), and the higher the monthly income, the lower the risk of MSDs (*OR* = 0.50, 95% *CI.*:0.34,0.74). Occupational stress (*OR* = 1.32, 95% *CI.*:1.05,1.67) and mental disorder(*OR* = 2.94, 95% *CI*.:2.25,3.84) increased the risk of MSDs. A Bayesian network diagram showed that occupational stress and MSDs have direct effects on mental disorders, and occupational stress can have indirect effects on mental disorders through MSDs.

**Conclusion:**

Our research shows that MSDs are common among coal miners. Occupational stress and psychological disorders can increase the incidence of MSDs.

## Background

With the development of industrialization and urbanization, great changes have taken place in labor production modes, and production operations are characterized by low labor load, high repetitive operation, high intensity working rhythms, and poor working posture. Traditional occupational health hazards have also shifted, creating a new range of occupational health problems such as musculoskeletal injuries, occupational stress, and psychological disorders [1, 2].

Musculoskeletal disorders (MSDs) are common skeletal injuries to muscles, bones, nerves, and other systems due to non-compliant working conditions; common skeletal injuries include lower back pain, cervical and shoulder wrist syndrome, and carpal tunnel syndrome [3, 4]. MSDs are very common occupational diseases in many industrialized countries, and they constitute one of the biggest occupational health burdens for modern occupational groups [5, 6]. As a common group of occupational diseases, MSDs have become the main cause of absenteeism and early retirement among the occupational population, as well as being a major risk factor for occupational disability [7]. The direct medical costs incurred by MSDs each year amount to tens of billions of dollars, due to sickness and absenteeism of employees [8]. MSDs occur in all labor groups in different industries. According to the different operation modes in different industries, prevalence, disease location, and other epidemic characteristics of MSDs are different in different industries. Industries with a high prevalence of MSDs include medical care, mining, the textile industry, construction, electronics, automobile manufacturing, and shipbuilding [9]. Studies have reported that the annual prevalence of MSDs in the medical care industry is as high as 29.0–74.0% [10–12], and the annual prevalence of MSDs in nurses is 43.1–80.8% [13]. According to a survey of construction workers, miners, transportation drivers and other occupational groups in the United States, Turkey, and the United Kingdom, the prevalence rate of lower back pain was 54.4–75.0% [14–16]. In addition to the direct losses caused by employee absenteeism, disease treatment and medical compensation, MSDs will also reduce the working efficiency and working ability of employees, and will affect their quality of life [17].

Occupational stress is a particular type of stress, which, unlike biological, physical, or chemical factors, can lead to specific occupational diseases, and can also damage health in non-specific ways [2]. The health effects of stress include physical, psychological, and behavioral changes [18]. One study has shown that occupational stress is an independent risk factor for MSDs, in addition to personal factors and ergonomic factors [19]. People with higher occupational stress tend to have more obvious responses to uncomfortable symptoms or pain, and higher occupational stress will affect their working ability and self-adjustment ability, thus increasing the risk of MSDs [20]. A clinical study has shown that occupational stress can affect the release of norepinephrine and epinephrine, and cause abnormal changes in heart rate and systolic blood pressure, thus affecting muscle activity [21].

The term mental disorder refers to a person who, due to physiological, psychological, or social reasons, or caused by a variety of abnormal psychological processes, shows abnormal personality characteristics or abnormal behavior [22]. According to the psychological theory, human behavior is controlled by individual psychological activities and is the external manifestation of individual psychological activities [23]. Studies have found that the root cause of unsafe behaviors in coal miners is an unhealthy mental state, and the level of mental health of coal miners has a significant predictive effect on the occurrence of accidents [24]. Among the SCL-90 factors of coal miners, the factors of compulsion, hostility, terror, paranoia, and psychosis are higher than the norm, the gap between the prevalence of psychotic factors in coal miners and normal people is the largest [25]. Perpetual excessive psychological pressure will reduce the mental health level of coal miners and cause psychosis [26]. Liu Yujiao [27] selected the research data of SCL-90 from 2007 to 2014 to investigate the mental health of coal miners and analyzed the scores of nine factors in 13,031 workers. They found that the scores of somatization and fear in coal miners were significant, suggesting that the mental health problems of coal miners in these two aspects were more prominent.

Researchers in many countries have carried out studies on the relationship between psychological factors and musculoskeletal disorders, but there is still no unified conclusion on the mechanism of the relationship between psychological factors and MSDs. Darvishi et al. [28] indicated that the risk of MSDs increased by 11.0% for every 1 unit of increase in psychological load level. Jingjing Wang et al. [29] found that psychological load has a direct effect on the occurrence of MSDs, and psychological load can also indirectly affect the occurrence of MSDs through posture. Habibi pointed out that poor psychological conditions can induce work stress, and work stress can increase muscle tension and shorten the interval of muscle activity, leading to muscle fatigue [30]. Research has found that work stress and negative mood will increase subjective sensitivity such as “pain”, leading to an increase in reports of MSDs. Meanwhile, via reverse causation, MSDs can also aggravate negative mood, leading to an increase in the incidence of stress, anxiety, and depression among employees [31].

Coal is China’s basic energy source, and China will continue to rely on coal as its main energy source for a long time in the future [32]. According to incomplete statistics, there are more than 6 million coal miners in China [33]. In coal mine production, given the different mining technologies, the harmful factors are varied; the working environment is often contaminated by dust, noise, and other occupational factors harmful to physical and mental health [34]. The underground working environment of a coal mine is also a relatively unique working environment: the underground working space is small, and the underground operators are not exposed to sunlight for extended periods of time. Mining itself and the transport and ventilation equipment are noisy and environmental humidity is high, so that coal mine workers are more prone to develop a variety of physiological and psychological problems [35]. One study found that coal miners had a high incidence of pneumoconiosis, the proportion of people with depressive symptoms was as high as 35.51%, and the proportion of people with job burnout was as high as 51.14% [36]. At the same time, owing to the cramped working space and high working intensity, the risk of coal miners suffering from MSDs increases [26]. Research has shown that more than 80% of social production accidents are caused unsafe behaviors [37], and 97.67% of major coal mine accidents are caused by human factors [38]. Therefore, controlling the unsafe behavior of coal miners, improving their mental health, reducing MSDs and injuries, and reducing the occurrence of accidents are key to ensuring the safety of coal production.

At present, most of the studies on coal miners focus on the harm caused by occupational characteristics and environmental factors to workers’ health, while few studies have focused on the relationship between psychological factors and MSDs. Therefore, this study conducted a cross-sectional survey on the occupational stress, mental health and MSDs of coal miners. We analyzed the risk factors for MSDs, and explored the relationships among occupational stress, mental health, and MSDs, so as to provide the basis for relevant departments to take effective measures to reduce the MSDs of coal miners and improve their mental health and quality of life.

## Methods

### Study population

From August 2018 to June 2019, stratified cluster random sampling was adopted. According to the annual output of their coal mines, coal mining companies are divided into large coal mining companies (annual output 1.2–3 million tons), medium-sized coal mining companies (annual output 300,000–1.2 million tons), and small coal mining companies (annual output less than 300,000 tons). All of the workers in two large, two medium, and two small coal mining companies (total six) were randomly selected as the research participants. The inclusion criteria were: coal miners aged from 18 to 60 with a working duration of > 1 year who volunteered to participate in this survey. A total of 1800 questionnaires were issued and 1675 valid questionnaires were recovered, with an effective questionnaire recovery rate of 93.06%. The research group established long-term cooperation with the respective local occupational disease hospitals. The investigators went to the location of each coal mine company with the medical examination team of the hospital. When the workers underwent occupational disease examination, the health questionnaire was distributed to them and the questionnaire was conducted. The study plan was approved by the Ethics Committee of Xinjiang Medical University, and all participants voluntarily signed informed consent before the investigation.

### Research methods

A questionnaire (detailed below) was used to investigate the status of occupational stress, MSDs, and mental health.

### General investigation

This section discusses general demographic characteristics such as sex, age, working years, educational level, marital status, night shift frequency, type of work, and monthly income.

### Occupational stress investigation

Occupational stress was elaborated using a self-reportable Chinese version of the Effort–Reward Imbalance (ERI) questionnaire proposed by Siegrist [39]. After testing, it is believed that the Chinese version of the ERI questionnaire has good reliability and validity, with a reliability of 0.91 and validity of 0.63 [40]. The questionnaire consists of three modules: effort, reward, and overload. The number of items in each module is 6, 11 and 6, respectively, with a total of 23 items. The Effort–Reward evaluation method is as follows: each item is given the same weight, and its index (ERI) = (11/6) × (E/R). ERI > 1 represents high effort–low reward, ERI = 1 represents effort–reward balance, and ERI < 1 indicates low effort–high reward. When ERI > 1, it is considered that there is occupational stress. The higher the ERI ratio, the higher the level of occupational stress.

### Mental health investigation

The Symptom Checklist 90 (SCL-90) compiled by Erogatis in 1975 was used to measure mental health [41]. The validity coefficient of each symptom in Chinese scale was 0.77–0.99 [42]. The scale includes 90 items, including somatization, compulsive symptoms, interpersonal sensitivity, depression, anxiety, hostility, fear, paranoia, psychosis, and another nine factors. Grades 1–5 were used: 1 = none, 2 = mild, 3 = moderate, 4 = heavy, and 5 = severe. The total score and factor score were used as indicators to evaluate the mental health level: the higher the score, the worse the mental health. Criteria for mental disorders were a total score ≥ 160 points or > 2 points for any factor.

### Musculoskeletal disorders investigation

The MSDs questionnaire jointly developed by Yang Lei et al. [43] was adopted, which was based on the Nordic Musculoskeletal Survey questionnaire [44]. This questionnaire was compiled based on national conditions in China and the research experience of domestic scholars, and is therefore suitable for evaluation of MSDs in the Chinese population. The overall Cronbach’sαcoefficient of the questionnaire was 0.81. The questionnaire had high structural validity, discriminant validity and predictive validity [45]. A total of nine body parts (neck, shoulder, back, waist, knee, ankle or foot, buttock or thigh, hand or wrist, and elbow) were included in this questionnaire. Each question was answered with “yes” or “no”. The respondents were asked whether they had any symptoms of discomfort in each body part in the past year. If the answer was yes, further inquiries were made as to absence from work as a result. A case was defined as the occurrence of musculoskeletal injury in one of the participants due to work reasons, when the duration of the symptoms was more than 24 h.

### Quality control

Selection and training of investigators: Each investigator underwent formal training to clarify the purpose and significance of the investigation, understand the principles and methods of the study design, and unify the meaning of indicators and requirements for questionnaire completion, so as to ensure the quality and progress of the investigation. In the field investigation, investigators conducted a comprehensive inspection of the contents of each completed questionnaire. If there was any doubt, the investigator asked for verification again; if there were any mistakes, the investigator corrected them and filled in the missing items. The questionnaire was input and checked independently by two investigators, and the relevant logic check was carried out. After data input, questionnaires were randomly selected at a rate of 20% for review, and statistical software was used to check consistency to ensure the accuracy of the database.

### Statistical analysis

SPSS for Windows v.22.0 software (SPSS Inc., Chicago, IL, USA) was used for statistical analysis of the data. Count data were statistically described by frequency and composition ratio, and the chi-square test was used for statistical analysis. Nonconditional logistic regression was used for multivariate analysis. R 3.6.1 software (Lucent Technologies., Jasmine Hill, New Jersey, USA) was used to write a Bayesian network program for model construction and analysis. The significance level was α = 0.05.

## Results

### The incidence of MSDs among coal miners with different demographic characteristics

Among the 1675 coal miners, 1471 were men and 204 were women. There were statistically significant differences in the prevalence of MSDs among coal miners of different sex, age, working years, shifts, type of work, educational level, and monthly income (*P* < 0.001). The prevalence of MSDs in females (73.0%) was higher than that in males (54.3%). The age group < 30 years had the lowest prevalence (34.0%), and the age group 50–60 years had the highest prevalence (74.3%). The prevalence of MSDs was lowest in the group with less than 5 years of work experience (43.4%), and highest in the group with > 15 years of work experience (70.8%). The prevalence was lower in the fixed day shift group (50.1%) and highest in the two shifts group (65.1%). Coal mine workers had the highest morbidity (68.2%), followed by safety supervisors (66.3%), while transportation workers had the lowest prevalence (37.5%). Regarding education level, the prevalence of MSDs was lowest in the junior college and above group (36.7%) and highest in the junior high school and below group (64.5%). The incidence of MSDs in the group with a monthly income of > 8001 was the lowest (39.7%), while the incidence of MSDs in the group with a monthly income of ≤5000 was the highest (59.8%) (Table 1).

**Table 1:** The incidence of MSDs among coal miners with different demographic characteristics [n (%)]

	Group	Number	MSDs(%)	*χ*^*2*^	*P*
Sex	Male	1471	799 (54.3)	25.565	< 0.001
	Female	204	149 (73.0)		
Age group, years	< 30	291	99 (34.0)	96.464	< 0.001
	30–39	464	259 (55.8)		
	40–49	667	402 (60.3)		
	50–60	253	188 (74.3)		
Working years	< 5	475	206 (43.4)	80.432	< 0.001
	5–15	649	352 (54.2)		
	> 15	551	390 (70.8)		
Shift	Fixed day shift	477	239 (50.1)	25.159	< 0.001
	Two shifts	538	350 (65.1)		
	Four shifts	660	359 (54.4)		
Type of work	Coal miner	330	225 (68.2)	107.562	< 0.001
	Heading driver	329	144 (43.8)		
	Transport worker	304	114 (37.5)		
	Move the frame worker	300	192 (64.0)		
	Safety supervisor	412	273 (66.3)		
Marital status	Single	282	167 (59.2)	0.950	0.330
	Married	1393	781 (56.1)		
Educational level	Junior High school and below	1004	648 (64.5)	86.299	< 0.001
	High school	300	164 (54.7)		
	Junior College and above	371	136 (36.7)		
Monthly income, yuan	≤5000	531	340 (64.0)	42.512	< 0.001
	5001–6500	415	248 (59.8)		
	6501–8000	490	265 (54.1)		
	>8001	239	95 (39.7)		

### The incidence of MSDs in different body parts among coal miners with different psychological disorders

There were statistically significant differences in the incidence of MSDs in all parts of the body of coal miners among groups with different psychological disorders (*P* < 0.001). The incidence of MSDs in all parts of the body of the mental disorder group was higher than that in the non-mental disorder group (Table 2).

**Table 2:** The incidence of MSDs in different parts of the body among coal miners with different mental disorders [n (%)]

Group	Neck	Shoul-der	Upper back	Elbow	Lower back	Wrist	Hip	Knee	Foot	MSDs
Mental disorder group	284 (44.9)	251 (39.7)	225 (35.5)	114 (18.0)	329 (52.0)	145 (22.9)	99 (15.6)	206 (32.5)	135 (21.3)	453 (71.6)
Non- Mental disorder group	246 (23.6)	218 (20.9)	153 (14.7)	78 (7.5)	329 (31.6)	110 (10.6)	66 (6.3)	171 (16.4)	82 (7.9)	495 (47.5)
*χ*^*2*^	82.266	68.532	98.074	42.973	68.712	46.538	38.400	58.760	63.241	92.791
*P*	< 0.001	< 0.001	< 0.001	< 0.001	< 0.001	< 0.001	< 0.001	< 0.001	< 0.001	< 0.001

### The incidence of MSDs among coal miners with different levels of occupational stress

There were statistically significant differences in the total incidence of MSDs and the incidence of MSDs in different body parts among coal miners in different occupational stress groups (*P* < 0.001). The incidence of MSDs in the occupational stress group was higher than that in the non-stress group (Table 3).

**Table 3:** The incidence of MSDs among coal miners with different levels of occupational stress [n (%)]

Group	Neck	Shoul-der	Upper back	Elbow	Lower back	Wrist	Hip	Knee	Foot	MSDs
ERI > 1	271 (35.6)	265 (34.8)	219 (28.8)	116 (15.2)	351 (46.1)	150 (19.7)	91 (12.0)	219 (28.8)	127 (16.7)	475 (62.4)
ERI ≤ 1	259 (28.3)	204 (22.3)	159 (17.4)	76 (8.3)	307 (33.6)	105 (11.5)	74 (8.1)	158 (17.3)	90 (9.8)	473 (51.8)
*χ*^*2*^	10.158	32.201	30.785	19.639	27.355	21.756	6.973	31.439	17.237	19.236
*P*	< 0.001	< 0.001	< 0.001	< 0.001	< 0.001	< 0.001	0.008	< 0.001	< 0.001	< 0.001

### Logistic regression analysis of factors influencing MSDs in coal miners

**Fig. 1:**
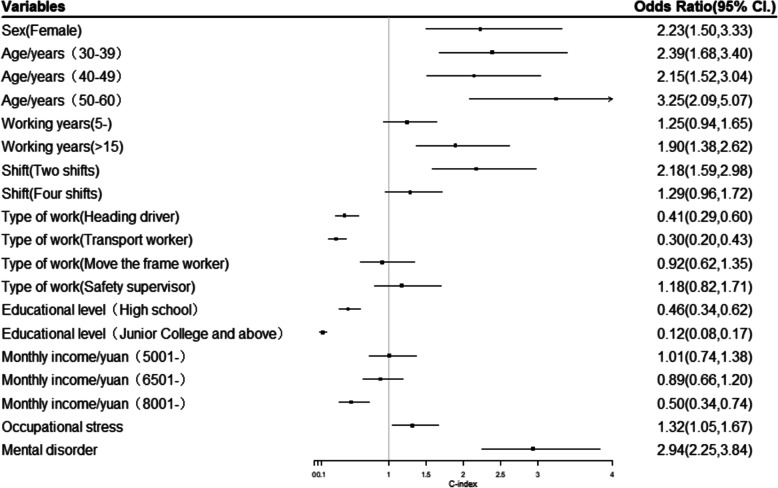
Logistic regression analysis of factors influencing MSDs in coal miners. Note: Independent variable assignment: Sex: male = 1, female =2; Age: < 30 = 1, 30–39 = 2, 40–49 = 3, 50–60 = 4; Working years: < 5 = 1, 5–15 = 2, > 15 = 3; Shift: fixed day shift = 1, two shifts = 2, four shifts = 3; Type of work: coal miner = 1, heading driver = 2, transport worker = 3, frame worker = 4, safety supervisor = 5; Education: junior high school or below = 1, high school = 2, junior college or above = 3; Monthly income: ≤5000 = 1, 5001- = 2, 6501- = 3, 8001- = 4; Occupational stress: non-occupational stress = 0, occupational stress = 1; Mental health status: non-mental disorders = 0, mental disorders = 1

MSDs was taken as the dependent variable, and individual factors with statistically significant differences (*P* < 0.05) in the univariate analysis (see Table 1) were used as the independent variables, as well as occupational stress and mental health, in the multivariate logistic regression analysis. The results showed that, compared with males, females had an increased risk of developing MSDs (*OR* = 2.23, 95% *CI*.:1.50,3.33). Compared with age < 30, the risk of MSDs was higher with other age groups (30-39 years:*OR* = 2.39, 95% *CI*.:1.68,3.40, 40-49 years:*OR* = 2.15, 95% *CI.*:1.52,3.04, 50-60 years:*OR* = 3.25, 95% *CI*.:2.09,5.07), and the longer the working years, the higher the risk of MSDs (*OR* = 1.90, 95% *CI*.:1.38,2.62). Workers who worked two shifts had an increased risk of developing MSDs compared with those who worked a fixed day shift (*OR* = 2.18, 95% *CI.*:1.59,2.98). Compared with coal miners, the risk of MSDs was lower in heading drivers and transport workers (*OR* = 0.41, 95% *CI.*:0.29,0.60, *OR* = 0.30, 95% *CI.*:0.20,0.43). The higher the education level, the lower the risk of MSDs (*OR* = 0.46, 95% *CI.*:0.34,0.62, *OR* = 0.12, 95% *CI.*:0.08,0.17), and the higher the monthly income, the lower the risk of MSDs (*OR* = 0.50, 95% *CI.*:0.34,0.74). Occupational stress and mental disorder increased the risk of MSDs (*OR* = 1.32, 95% *CI.*:1.05,1.67, *OR* = 2.94, 95% *CI*.:2.25,3.84) (Fig. 1).

### Relationship model of mental disorder, occupational stress, and MSDs constructed by Bayesian network analysis

**Fig. 2:**
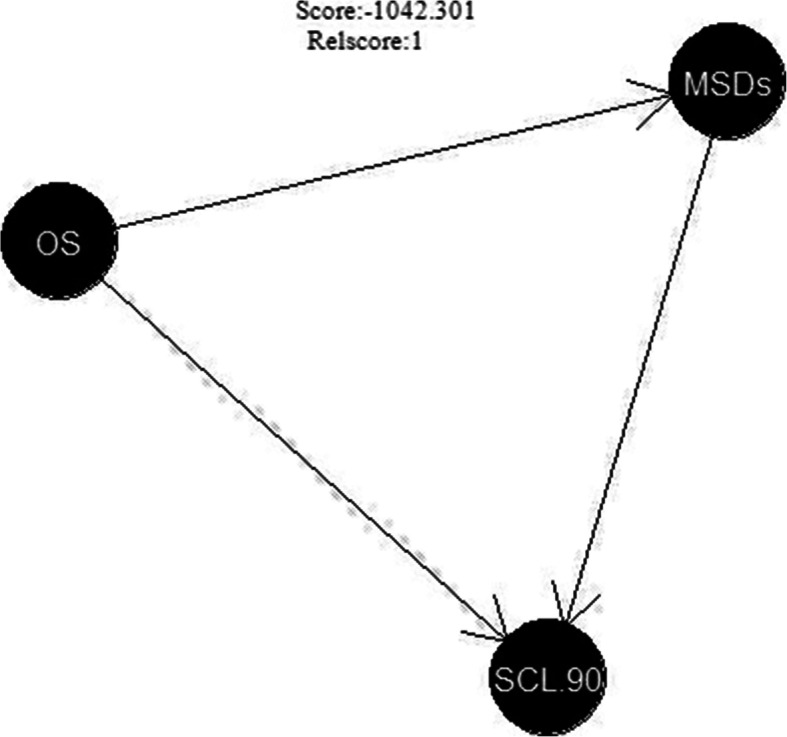
Bayesian network diagram of mental disorders, occupational stress, and MSDs. Note: OS = Occupational stress; MSDs = Musculoskeletal disorders; SCL.90 = mental disorder

A Bayesian network model was used to analyze further the relationships between psychological disorders, occupational stress, and MSDs. Occupational stress, mental disorder, and MSDs were set as latent variables, and the indicators of the three scales were taken as observational variables. R software was used to write the programs and build the Bayesian network diagram. It can be seen from the figure that occupational stress and MSDs have direct effects on mental disorders, and occupational stress can have indirect effects on mental disorders through MSDs (Fig. 2).

## Discussion

The harm of MSDs is increasingly significant, and MSDs have become the second leading cause of disability in the world [46]. Studies have demonstrated that mental illness and MSDs have a high prevalence among coal miners, and are the main cause of absenteeism and disability among coal miners [47, 48].

This survey found that the prevalence of MSDs in women was higher than in men; this may be because the working environment in the coal mine is harsh and the labor intensity is high, and as women’s physical strength is not as high as men’s, coal mining may not be suitable for females. Therefore, the prevalence of MSDs in female miners was higher than that in male miners [49]. The prevalence of MSDs in all body parts of miners in the groups with age < 30 years and working years < 5 years was the lowest; the prevalence increased with the increase of age and working years, which was consistent with the research results of Lei et al. [50]. Younger miners are generally in good physical condition, have a better ability to resist external damage and repair themselves, and are less likely to detect diseases. However, with the increase of age, injuries to all parts of the body gradually accumulate with the increase of labor intensity, leading to the gradual increase in the prevalence of MSDs in miners. The prevalence of MSDs was highest in miners with two shifts. Compared with other shift miners in the two shift group have a longer working hours,so they have higher degree of musculoskeletal fatigue and cannot get sufficient rest. As a result, muscle fatigue gradually accumulates, leading to the occurrence of MSDs [51]. Coal miners have the highest prevalence of MSDs. The reason is related to the low degree of mechanization of coal mining work, high labor intensity, relatively narrow working space, frequent need to lift weights during work, and poor posture for operations and other factors. The prevalence of MSDs was highest in miners with junior middle school education and below, and the prevalence decreased with the increase of education level. Kiadaliri et al. [52] drew similar conclusions. Miners with lower education levels are generally engaged in heavy physical labor underground, and their labor intensity is higher than that of other workers working underground, so they are more prone to muscle fatigue and injury, leading to a higher prevalence of MSDs. It is suggested that management should carry out health education to improve workers’ awareness of health care, and arrange workers’ rotation time reasonably, so that workers can have enough time to relieve muscle fatigue and recover their physical strength [53]. The prevalence of MSDs in all body parts was highest in the group with monthly income ≤5000, and the prevalence decreased with the increase of monthly income. People with low salaries may be more willing to work hard, even overtime, to obtain higher labor remuneration. Their work intensity, working hours, and working pressure are all higher than those with high incomes, and MSDs are more likely to occur. In addition, people with higher income from work may invest more in their own health care and may also therefore have a lower prevalence of MSDs.

The prevalence of MSDs in the group of coal miners with occupational stress was higher than that in the non-stress group, and the prevalence of MSDs in the mental disorder group was higher than that in the non-mental disorder group, suggesting that occupational stress and mental disorder may increase the prevalence of MSDs. The results of the logistic regression analysis showed that occupational stress and mental disorder were risk factors for MSDs in coal miners. Occupational stress is a physiological and psychological reaction caused by long-term and chronic stress during specific working conditions. Occupational stress can cause changes in nerves, hormones, and blood pressure, leading to enhanced musculoskeletal co-activation, thereby increasing musculoskeletal system load and inducing or aggravating MSDs. Therefore, occupational stress is considered as an important cause of MSDs [54]. Jay et al. [55] believe that, when work demands are high, the risk of neck, shoulder, and waist joint pain will be significantly increased. Herr et al. [56] believe that occupational stress can lead to enhanced muscle co-activation, and thus to increased musculoskeletal system load, inducing or aggravating MSDs. According to Faoro et al. [57], the risk of MSDs in employees with mental disorders is twice that of workers without mental disorders, and the effect of mental disorders is still significant after adjusting for sociodemographic, behavioral, health-related, and occupational variables. Coal miners are away from home for extended periods of time, which causes increased psychological and emotional strain. In addition, there are few psychological counseling interventions specifically aimed at coal miners, so a psychological stress reaction occurs readily in this group, which will further affect physical health and lead to the occurrence and development of MSDs. At the same time, coal miners have heavy working tasks and high working pressure, and work overload will cause persistent pain in all parts of the body [58]. A clinical study showed that occupational stress is common among steel workers; it is closely related to musculoskeletal pain, and may be associated with the occurrence and development of MSDs [59]. If the high-pressure working environment and long-term continuous tension reaction cannot be effectively alleviated, it is easy to generate various psychological problems and develop serious mental disorders [60]. The occurrence of psychological problems will increase the risk of MSDs [61]. Studies have found that pain and negative emotions coexist: more than half of the patients with mental diseases have concurrent MSDs, and psychological problems will increase the probability of MSDs by a factor of two when compared with healthy people [62]. A Bayesian network model was used to analyze further the relationships between occupational stress, mental disorder and MSDs. The results show that occupational stress has a direct effect on MSDs, occupational stress and MSDs have a direct effect on mental disorders, and occupational stress can have an indirect effect on mental disorders through MSDs. Research indicates that occupational stress is one of the risk factors for MSDs [63]. Mental load has an influence on fatigue and physical recovery, which are closely related to the development of MSDs. Mental stress and tension have adverse effects on biomechanical responses. Stress affects movement and gait control, further disturbs joint stability, and leads to joint contraction via compensatory muscles and increased muscle tension [64]. In addition, mental stress can cause continuous secretion of catecholamines and cortisol, which may hinder musculoskeletal recovery [65]. At the same time, via reverse causation, MSDs can lead to negative emotions, aggravating mental health problems [66]. As a result of the pain in their bodies, laborers cannot rest sufficiently after working, which produces negative moods such as anxiety and agitation, thus increasing the risk of occurrence of psychological disorders. MSDs may cause negative interference in social activities and psychology, further increasing the risk of mental problems, therefore, a two-way connection and feedback loop effect exist between the two [63]. Strengths in this study included a sufficient sample size and the fact that the reliability and validity of the international questionnaire were robust. However, this study was a preliminary study, which concluded that occupational stress and mental disorder increase the risk of MSDs, and further research on the mechanism of these three factors should be conducted in the future to link the interaction between genes and environment to the influence of MSDs.

## Conclusion

Our study found that, due to the unique working environment, coal miners have a high prevalence of MSDs. In addition to demographic characteristics, occupational stress, and mental disorders are also risk factors for MSDs. A Bayesian network model was used to analyze further the relationships between occupational stress, mental disorders and MSDs. The results show that occupational stress has a direct effect on MSDs, occupational stress and MSDs have a direct effect on mental disorders, and occupational stress can have an indirect effect on mental disorders through MSDs. Therefore, reducing workers’ stress level and improving their mental health status can alleviate the musculoskeletal injury of workers.

## Data Availability

The datasets used and analyzed during the current study are available from the corresponding author on reasonable request.

## Abbreviations

MSDs: Musculoskeletal disorders
ERI: Effort-Reward Imbalance
SCL-90: Symptom Checklist-90

## References

[1] Soteriades ES, Psalta L, Leka S, Spanoudis G (2019). Occupational stress and musculoskeletal symptoms in firefighters. Int J Occup Med Environ Health.

[2] Moreno Fortes A, Tian L, Huebner ES (2020). Occupational stress and employees complete mental health: a cross-cultural empirical study. Int J Environ Res Public Health.

[3] Kaliniene G, Ustinaviciene R, Skemiene L, Januskevicius V (2013). Associations between neck musculoskeletal complaints and work related factors among public service computer workers in Kaunas. Int J Occup Med Environ Health.

[4] Gibson DS, Rooney ME (2016). Musculoskeletal diseases. Proteomics. Clin Appl.

[5] Mohammadipour F, Pourranjbar M, Naderi S, Rafie F (2018). Work-related musculoskeletal disorders in Iranian office workers: prevalence and risk factors. J Med Life.

[6] Tuček M, Vaněček V (2020). Musculoskeletal disorders and working risk factors. Cent Eur J Public Health.

[7] Weerasekara I, Hiller CE (2017). Chronic musculoskeletal ankle disorders in Sri Lanka. BMC Musculoskelet Disord.

[8] Bhattacharya A (2014). Costs of occupational musculoskeletal disorders (MSDs) in the United States. Int J Ind Ergon.

[9] Bernard BP (1997). Musculoskeletal disorders and workplace factors: a critical review of epidemiologic evidence for work-related musculoskeletal disorders of the neck, upper extremity, and low back.

[10] Kee D, Seo SR (2007). Musculoskeletal disorders among nursing personnel in Korea. Int J Ind Ergon.

[11] Lipscomb J, Trinkoff A, Brady B, Geiger-Brown J (2004). Health care system changes and reported musculoskeletal disorders among registered nurses. Am J Public Health.

[12] Mehrdad R, Dennerlein JT, Haghighat M, Aminian O (2010). Association between psychosocial factors and musculoskeletal symptoms among Iranian nurses. Am J Ind Med.

[13] Zhang L, Cui C, Wang J (2018). Overview of prevalence and risk factors of work-related musculoskeletal disorders in nursing staff. J Mod Prev Med.

[14] Robb M, Mansfield NJ (2007). Self-reported musculoskeletal problems amongst professional truck drivers. Ergonomics.

[15] Widanarko B, Legg S, Stevenson M, Devereux J, Jones G (2013). Prevalence of low back symptoms and its consequences in relation to occupational group. Am J Ind Med.

[16] Boschman JS, Fringsdresen MH, Van HDM (2015). Use of ergonomic measures related to musculoskeletal complaints among construction workers: a 2-year follow-up study. Saf Health Work.

[17] Ge H, Sun X, Liu J (2018). The status of musculoskeletal disorders and its influence on the working ability of oil workers in Xinjiang, China. Int J Environ Res Public Health.

[18] Faraji A, Karimi M, Azizi SM, Janatolmakan M, Khatony A (2019). Occupational stress and its related demographic factors among Iranian CCU nurses: a cross-sectional study. BMC Res Notes.

[19] Hämmig O (2020). Work- and stress-related musculoskeletal and sleep disorders among health professionals: a cross-sectional study in a hospital setting in Switzerland. BMC Musculoskelet Disord.

[20] Van Eerd D, Munhall C, Irvin E, Rempel D, Brewer S, van der Beek AJ, Dennerlein JT, Tullar J, Skivington K, Pinion C, Amick B (2016). Effectiveness of workplace interventions in the prevention of upper extremity musculoskeletal disorders and symptoms: an update of the evidence. Occup Environ Med.

[21] Ning L, Zhang Y, Guan S (2017). Analysis of correlation between musculoskeletal disorders and sleep quality and occupational stress in medical staff in a hospital. Chin J Occup Med.

[22] Steel Z, Marnane C, Iranpour C, Chey T, Jackson JW, Patel V, Silove D (2014). The global prevalence of common mental disorders: a systematic review and meta-analysis 1980-2013. Int J Epidemiol.

[23] Schaefer JD, Caspi A, Belsky DW, Harrington H, Houts R, Horwood LJ, Hussong A, Ramrakha S, Poulton R, Moffitt TE (2017). Enduring mental health: prevalence and prediction. J Abnorm Psychol.

[24] Considine R, Tynan R, James C, Wiggers J, Lewin T, Inder K, Perkins D, Handley T, Kelly B (2017). The contribution of individual, social and work characteristics to employee mental health in a coal mining industry population. PLoS One.

[25] Jian Y, Yuqing X, Nurlan MV (2020). Investigation on occupational stress and mental health of 491 coal miners. J Xinjiang Med Univ.

[26] Liu L, Wen F, Xu X, Wang L (2015). Effective resources for improving mental health among Chinese underground coal miners: perceived organizational support and psychological capital. J Occup Health.

[27] Liu YJ, Liu J, Zhu BY (2018). Mental health status of Chinese underground coal mine workers from 2007 to 2014. J Saf Environ.

[28] Darvishi E, Maleki A, Giahi O, Akbarzadeh A (2016). Subjective mental workload and its correlation with musculoskeletal disorders in bank staff. J Manip Physiol Ther.

[29] Wang J, Cao Y, Jin X (2018). Analysis of influencing factors of neck musculoskeletal disorders among porters in an airport. Chin J Occup Med.

[30] Habibi E, Taheri MR, Hasanzadeh A (2015). Relationship between mental workload and musculoskeletal disorders among Alzahra hospital nurses. Iran J Nurs Midwifery Res.

[31] Lee JE, Watson D, Frey LA. Psychological factors predict local and referred experimental muscle pain: A cluster analysis in healthy adults. European journal of pain (London, England). 2013;17(6):903–15. 10.1002/j.1532-2149.2012.00249.x PMC3594074 23165778

[32] Dong S, Xu B, Yin S, Han Y, Zhang X, Dai Z (2019). Water resources utilization and protection in the coal mining area of northern China. Sci Rep.

[33] Han S, Chen H, Harvey MA, Stemn E, Cliff D (2018). Focusing on coal Workers' lung diseases: a comparative analysis of China, Australia, and the United States. Int J Environ Res Public Health.

[34] Lu Y, Zhang Z, Gao S, Yan H, Zhang L, Liu J (2020). The status of occupational burnout and its influence on the psychological health of factory workers and miners in Wulumuqi, China. Biomed Res Int.

[35] Liu L, Wang L, Chen J (2014). Prevalence and associated factors of depressive symptoms among Chinese underground coal miners. Biomed Res Int.

[36] Yu M, Duan XX, Chen SM (2015). Predictive analysis of safety psychology and mental health of coal miners on safety accidents. Chin J Health Psychol.

[37] Jiang JC (2004). Accident investigation and analysis technology.

[38] Wang JF, Li WJ (2002). Coal Mine Accidents and Expert Comments in China (the first volume).

[39] Siegrist J, Wege N, Puhlhofer F (2009). A short generic measure of work stress in the era of globalization: effort-reward imbalance. Int Arch Occup Environ Health.

[40] Dai J, Yu H, Wu J (2007). A concise occupational stress questionnaire development and evaluation model construction. J Fudan Univ (Medical Edition).

[41] Wang X, Wang X, Ma H (1999). Manual of mental Health Rating Scale.

[42] Su Q, Liu Y, Chen Y (2012). Study on reliability and validity of hospital anxiety and depression scale applied in health examination center. Sichuan Med.

[43] Yang L, Hildebrandt VH, Yu S (2009). An introduction to the musculoskeletal disorders questionnaire is attached. J Ind Hyg Occup Dis.

[44] Romaguera D, Angquist L, Du H (2010). Dietary determinants of changes in waist circumference adjusted for body mass index-aproxy measure of visceral adiposity. PLoS One.

[45] Du W, Wang S, Wang J (2012). Evaluation of reliability and validity of the musculoskeletal disorders questionnaire. Chin J Occup Health Labor.

[46] Vos T, Flaxman AD, Naghavi M (2012). Years lived with disability (ylds) for 1160 sequelae of 289 diseases and injuries 1990-2010: a systematic analysis for the global burden of disease study 2010. Lancet (London, England).

[47] Deng M, Wu F, Wang J (2017). Musculoskeletal disorders, personality traits, psychological distress, and accident proneness of Chinese coal miners. Work (Reading, Mass.).

[48] Walter AW, Morocho C, King L, Bartlett J, Kelsey D, DeSousa M, Biesecker G, Punnett L (2018). Preventing opioid use disorders among fishing industry workers. Int J Environ Res Public Health.

[49] Ge H (2018). Occupational epidemiology of copper and nickel miners and their unsafe behaviors.

[50] Lei L, Li Y, Liu X (2019). Analysis on the present situation and influencing factors of occupational musculoskeletal disorders among nurses in a hospital in Luzhou city. J Ind Hyg Occup Dis.

[51] Yong X, Li F, Ge H, Sun X, Ma X, Liu J (2020). A cross-sectional epidemiological survey of work-related musculoskeletal disorders and analysis of its influencing factors among coal mine workers in Xinjiang. Biomed Res Int.

[52] Kiadaliri AA, Petersson IF, Englund M (2019). Educational inequalities in mortality associated with rheumatoid arthritis and other musculoskeletal disorders in Sweden. BMC Musculoskelet Disord.

[53] Wang T, Zhao YL, Hao LX, Jia JG (2019). Prevalence of musculoskeletal symptoms among industrial employees in a modern industrial region in Beijing, China. Chin Med J.

[54] Hao X, Xu H, Shi S, Li Q (2020). A survey of occupational musculoskeletal disorders and Occupational stress in steel workers. J Ind Hyg Occup Dis.

[55] Jay K, Andersen LL (2018). Can high social capital at the workplace buffer against stress and musculoskeletal pain cross-sectional study. Medicine (Baltimore).

[56] Herr RM, Bosch JA, Loerbroks A, van Vianen AEM, Jarczok MN, Fischer JE, Schmidt B (2015). Three job stress models and their relationship with musculoskeletal pain in blue-and white-collar workers. J Psychosom Res.

[57] Faoro MW, Olinto MTA, Paniz VMV, Macagnan J, Henn RL, Garcez A, Pattussi MP (2018). Work-related musculoskeletal pain and its association with common mental disorders among employees of a poultry producing company in southern Brazil. Rev Bras Med Trab.

[58] Miranda H, Viikari-juntura E, Martikainen R (2001). A prospective study of work related factors and physical exercise aspredictors of shoulder pain. Occup Environ Med.

[59] Wang J, Kang H, Chang Q (2019). Distribution characteristics of occupational-disease-inductive factors in newly added enterprises in Yulin, Shaanxi Province in 2017. Med Anim Control.

[60] De Simone S, Cicotto G, Lampis J (2016). Occupational stress, job satisfaction and physical health in teachers. Revue Europeenne De Psychologie Appliquee.

[61] Ng YM, Voo P, Maakip I (2019). Psychosocial factors, depression, and musculoskeletal disorders among teachers. BMC Public Health.

[62] Korniloff K, Kotiaho S, Vanhala M (2017). Musculoskeletal pain in melancholic and atypical depression. Pain Med (Malden, Mass.).

[63] Han F, Wang D, Zhou J (2017). Research progress on the influence of occupational stress on work-related musculoskeletal disorders. Chin J Occup Med.

[64] Mehta RK, Agnew MJ (2012). Influence of mental workload on muscle endurance, fatigue, and recovery during intermittent static work. Eur J Appl Physiol.

[65] Almeida LB, Vieira ER, Zaia JE (2017). Musculoskeletal disorders and stress among footwear industry workers. Work (Reading, Mass.).

[66] Xu G, Li L, Liu F (2012). Study on the relationship between musculoskeletal injury and psychosocial factors in coal miners. Chin J of Occup Health Labor.

